# We, Them, and It: Dictator Game Offers Depend on Hierarchical Social Status, Artificial Intelligence, and Social Dominance

**DOI:** 10.3389/fpsyg.2020.541756

**Published:** 2020-11-23

**Authors:** Martin Weiß, Johannes Rodrigues, Marko Paelecke, Johannes Hewig

**Affiliations:** Department of Psychology I: Differential Psychology, Personality Psychology and Psychological Diagnostics, Institute of Psychology, University of Würzburg, Würzburg, Germany

**Keywords:** decision-making, dictator game, personality, social dominance, social status

## Abstract

We investigated the influence of social status on behavior in a modified dictator game (DG). Since the DG contains an inherent dominance gradient, we examined the relationship between dictator decisions and recipient status, which was operationalized by three social identities and an artificial intelligence (AI). Additionally, we examined the predictive value of social dominance orientation (SDO) on the behavior of dictators toward the different social and non-social hierarchical recipients. A multilevel model analysis showed that recipients with the same status as the dictator benefited the most and the artificial intelligence the least. Furthermore, SDO, regardless of social status, predicted behavior toward recipients in such a way that higher dominance was associated with lower dictator offers. In summary, participants treated other persons of higher and lower status equally, those of equal status better and, above all, an algorithm worst. The large proportion of female participants and the limited variance of SDO should be taken into account with regard to the results of individual differences in SDO.

## Introduction

In different contexts of life, people repeatedly encounter situations in which they act as either dominant or subordinate, for example, when an employee has to obey his or her superior or when parents order their child to do his or her homework. To address such situations, Sidanius and Pratto (1999) postulated the social dominance theory (SDT) and created a comprehensive model of social relations. In addition to the core aspects of SDT, hierarchy and dominance, several factors are considered important, such as race, gender, age, and religion. Pratto et al. (2006) explained that the basis for group-level hierarchies covers several levels, including individual, group, and institutional behavior.

In the present study, we focus on the influence of the social status of the recipient on the decisions of the dictator in the dictator game (DG; Forsythe et al., 1994). The classic DG is an anonymous, one-shot decision-making task in which one party, the dictator, is provided with an endowment. The dictator is asked to divide this endowment between himself and a partner, the so-called recipient. The DG thus reflects an assessment of altruistic versus selfish behavior in a hierarchical social interaction, since the recipient has no scope for action. Ball et al. (2001) showed in a study on the economic benefits of experimentally manipulated status that members of the high-status group received lucrative offers. Similarly, Glaeser et al. (2000) reported that participants traded favorably in a trust game with individuals of high status. Their study suggested a more trustworthy behavior of the trustee toward participants with high status. In a dictator game study, Rodrigues et al. (2015) were able to show that income as a possible proxy for social status, significantly influenced dictator offers. Low-income recipients received the most generous offers and high-income recipients, the lowest. Finally, Liebe and Tutic (2010) have investigated how status (operationalized with the affiliation to different types of German schools) influences the behavior of dictators and recipients. The authors demonstrated that higher status of the dictators was associated with higher offers and that on the recipient side, higher status was associated with lower offers. Since this experiment was limited to 14- to 18-year-old participants in schools, we wanted to enhance this research by a study in adult life. In addition, the school children were tested in a between-subject design, while in the present experiment, a different approach was applied based on the dictator’s behavior in a within-subject design toward different social hierarchies. Another influential factor in social behavior that should not remain unmentioned is affect. Negative affect increased dictator allocations compared to positive affect, since negative affect led to a stronger external orientation with more concerns about social norms (Forgas and Tan, 2013). Capraro (2019) pointed out in his review on the cognitive basis of social behavior that different emotions influence social decisions in different ways. The consequence is that emotionally driven decisions based on a particular emotion do not necessarily lead to rapid and default processing. Since social hierarchies include differences in status and can also have emotional connotations, social decisions in a hierarchical structure can be very complex for the individual.

As a novel contribution, we also investigated whether social dominance can be perceived exclusively as a social phenomenon. Recently, Melo et al. (2016) had subjects play several social bargaining games and instructed them that they would play against either a human or against a computer. The authors were able to show that human beings would not feel guilty if they exploited machines. De Kleijn et al. (2019) examined the influence of anthropomorphizing various opponents in the ultimatum and dictator game. They were able to show that fairness concerns in the ultimatum game were not influenced by the physical appearance of the opponent, but by individual differences in the anthropomorphization of others. Regarding altruistic behavior in the dictator game, however, an influence of physical appearance was found. A humanoid robot achieved the lowest dictator offers compared to technical-looking robots. For this reason, we have introduced a bot as one of the recipients in the design of our study. We wanted to clarify whether this opponent is treated similarly to certain hierarchy levels in a game framed by social status.

The personality trait that corresponds to the social dominance theory is the so-called social dominance orientation (SDO). Sidanius and Pratto (1999, p. 61) specified this as follows:

Social dominance orientation is defined as a very general individual difference orientation expressing the value that people place on non-egalitarian and hierarchically structured relationships among social groups. It expresses general support for the domination of certain socially structured groups over other[s]… regardless of the manner in which these groups are defined. These groups may be defined on the basis of race, sex, nationality, ethnicity, religion, social class, region, skin color, caste, lineage, tribe, minimal groups, or any other group distinction that the human mind is capable of constructing. Individuals differ in the degree to which they desire group-based inequality and dominance […].

In summary, SDO distinguishes people according to their striving for hierarchy, dominance, and inequality between groups at several levels. Consequently, SDO also reflects the extent to which individuals support the dominance of parent groups over subordinate groups. Other authors (e.g., Duckitt et al., 2002) refer SDO to the personality dimensions of “toughmindedness” and “tendermindedness.” Therefore, individuals who score high on SDO may perceive their world as a competition in which the pursuit of dominance is crucial. In comparison, individuals who score low on SDO believe in cooperation and harmony.

Concerning the competitive world view, several studies have shown that high SDO is associated with certain social attitudes, such as in-group discrimination (Pratto et al., 1994), hostile sexism and racism (Nicol and Rounding, 2013), or generally negative attitudes toward different groups (e.g., Altemeyer, 1998). Focusing on social economic bargaining, the literature review has not identified any relevant studies that have established a link between social dominance orientation and prosocial behavior. Only a recently published study could show, by analyzing questionnaire data based on a Chinese sample, that SDO is negatively related to prosocial behavior (and subjective well-being). This negative correlation was even stronger among women than among men (Yang et al., 2019).

We propose that the DG is a situation where socially dominant personality traits are activated, leading to the exploitation of the dominant position. According to the trait activation theory (Tett and Burnett, 2003), traits are expressed when a particular situation contains trait-relevant cues. Furthermore, it is still unclear in the literature whether social dominance also manifests itself in the face of artificial algorithms or is a personality trait characterized exclusively by social traits.

In summary, the aim of this study is to examine the influence of different hierarchical levels on the distribution of money in the dictator game, as well as the moderating role of the personality trait social dominance orientation. Based on the literature, we assume that artificial intelligence is treated as a non-social interaction partner in a particularly unfairly and dehumanized way and consequently receives the lowest offers regardless of social dominance. Furthermore, we assume that higher social dominance leads to lower offers for socially lower ranked individuals and to higher offers for higher ranked individuals.

## Method

### Ethics Statement

The study was carried out in accordance with the recommendations of “Ethical Guidelines, The Association of German Professional Psychologists” (“Berufsethische Richtlinien, Berufsverband Deutscher Psychologinnen und Psychologen”). All participants gave informed consent in accordance with the Declaration of Helsinki before they participated in the experiment. During the experiment, a cover story was used, but participants were informed about this deception as soon as the task was over, as is common practice in psychological experiments.

### Participants

The experiment was created with the online questionnaire platform SoSci Survey (Leiner, 2019) and made available to the participants on www.soscisurvey.de. Of 102 participants who started the experiment, 7 did not complete the entire task, so that the final sample for data analysis included 95 participants (91% female; *M*_age_ = 22.21, *SD*_age_ = 7.09). The participants received either course credit or a monetary compensation of 5€.

### Experimental Procedure

First, participants were asked to complete several questionnaires on demography and personality. These included the 16 items of the *Social Dominance Orientation Scale* (SDO-7; Saldarriaga et al., 2017) for assessing social dominance. For explorative purposes, we also assessed the German version of the International Personality Item Pool (Johnson, 2014). The participants were also asked to indicate whether they were studying or working with the aim of adapting the following experiment to their social situation. Subsequently, the participants played the role of the dictator in a dictator game against four different identities. The participants were asked to make five offers per identity by selecting pi-charts that represented offers of 0, 1, 2, 3, 4, and 5 € for an identity that is considered in the social hierarchy as lower, one as equal, one as higher, and finally one identity that is represented by an artificial intelligence (AI). From the perspective of a student, these identities were represented as a high school graduate (lower), a doctoral student (higher), another student (equal), or AI. From the perspective of a worker, these identities were represented as an apprentice (lower), an executive (higher), another worker (equal), or AI. The identities were presented in a within-subject design and in blocks in randomized order.

### Statistical Analysis

We used R software (R Core Team, 2019) and the nlme package (Pinheiro et al., 2020), with the procedure “lme” and the psych package (Revelle, 2020), to statistically analyze our data by two-level hierarchical linear modeling. We were particularly interested in the *offered amount of money* (level-1 outcome variable), the *opponent* for whom the offer was made (level-1 predictor variable; factor with four levels: equal status, higher status, lower status, and AI), and the individual *score on social dominance orientation* (level-2 predictor variable; grand mean centered). We included both predictors and the cross level interaction of *opponent* and *social dominance orientation* into our model and random slopes for the factor *opponent*. The model fit was determined using the Akaike information criterion (AIC), as displayed in Table 1. To investigate the difference between the social and non-social opponents in detail, we performed Helmert contrasts in the mixed model, resulting in the following contrasts: human (equal, higher, and lower status) versus AI, equal status versus non-equal status (higher and lower status) and higher versus lower status. To fully examine the influence of social status, we used Bonferroni adjusted *post hoc* comparisons.

**TABLE 1:** Akaike Information Criterion (AIC) for linear mixed-effects models and information loss difference.

Model	AIC	Δ (AIC)	*p* (information loss)
Baseline model	7,689.83	3,026.17	<0.001
Model with level 1 predictor	6,123.86	1,460.2	<0.001
Model with level 1 and level 2 predictors	4,663.66		

*The first column indicates the selected model. Afterward, the AIC value and the relative information loss of the baseline model and the model with level 1 predictors compared to the model with level 1 and level 2 predictors is displayed. The last column indicates *p-*values of the model comparisons.*

## Results

The regression coefficient relating the dictator offers to AI versus human interaction partners was negative and statistically significant (β = −2.48, *p* < 0.001), as shown in Table 2. The comparison of equal to unequal status was also statistically significant (β = 0.52, *p* < 0.001), whereas high and low status did not differ (β = 0.11, *p* = 0.439).

**TABLE 2:** Results of the hierarchical linear model analysis.

Fixed effect	Coefficient	SE	*t*-value	*p*-value
Intercept	3.19	0.09	34.98	<0.001
AI vs. human	–2.48	0.17	–14.51	<0.001
Equal vs. high and low	0.52	0.09	5.66	<0.001
High vs. low	–0.11	0.14	–0.77	0.439
SDO	–0.45	0.11	–4.09	<0.001
AI vs. human × SDO	0.29	0.21	1.40	0.162
Equal vs. high and low × SDO	–0.06	0.11	–0.52	0.603
High vs. low × SDO	–0.08	0.18	–0.46	0.648

*The beta coefficient, standard errors of the mean, *t*-values, and *p*-values are displayed separately for main effects and interactions.*

The main effect of social dominance was also significant (β = −0.45, *p* < 0.001), indicating a decrease in dictator offers with an increase in social dominance orientation. The cross−level interaction between the opponent and social dominance was not significant for all contrasts (AI vs. human: β = 0.29, *p* = 0.162; equal vs. high and low: β = −0.06, *p* = 0.603; high vs. low: β = −0.08, *p* = 0.648).

**FIGURE 1:**
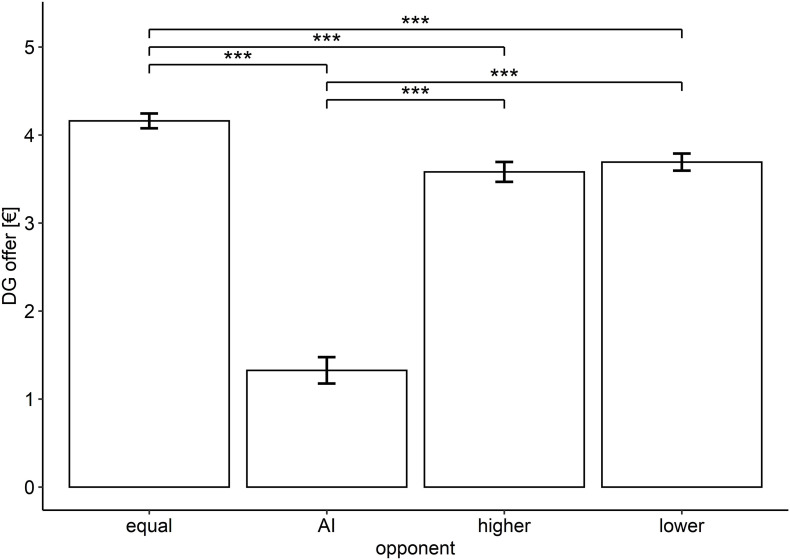
Effect of the different opponents on dictator offers. The error bars indicate the standard error of the mean, ^∗∗∗^*p* ≤ 0.001.

The *post hoc* comparisons of the four opponents indicated that all of the three social identities received significantly more money compared to the AI identity (all values of *p* < 0.011; *M_*equal*_* = 4.16, *M_*higher*_* = 3.58, *M_*lower*_* = 3.69; *M_*AI*_* = 1.32). Furthermore, opponents of both lower and higher status received less money compared to the equally framed opponent (*p* < 0.001). Finally, lower- and higher-framed opponents did not differ in their received money (*p* = 0.438), as illustrated in Figure 1. To test for the consistency of the dictator decisions toward the four opponents, we calculated an intraclass coefficient (ICC). The ICC (*k* = 95 raters) was based on a two-way mixed-effects model and revealed a measure ICC of 0.56. Hence, we found a moderate degree of consistency for the dictator decisions across the different recipients. As illustrated in Table 3, the social opponents correlated highly with each other (all values of *r* ≥ 0.742), but not with AI (all values of *r* ≤ 0.196). Moreover, we compared the correlation coefficients (Eid et al., 2011) between SDO and each of the four opponents. The correlation coefficients between SDO and equal status compared to SDO and AI differed significantly (*p* = 0.018). By trend, the coefficients between SDO and equal status compared to SDO and lower status (*p* = 0.061) and the coefficients between SDO and higher status compared to SDO and AI (*p* = 0.08) were also different. The other comparisons did not yield significance (all values of *p* ≥ 0.12). For exploratory reasons, we performed correlations of the subscale *altruism* of the International Personality Item Pool with SDO, the four identities and the dictator offers averaged across all trials (see Table 3). While dominance and altruism did not correlate significantly (*r* = −0.114, *p* = 0.273), higher scores on altruism led to higher average dictator offers (*r* = 0.215, *p* = 0.036). Especially, the identity of lower status received more money from altruists (*r* = 0.243, *p* = 0.018).

**TABLE 3:** Confirmatory and exploratory correlations for the average amount of dictator offers across all trials, the offers toward the four identities, social dominance orientation (SDO), and trait altruism.

	Confirmatory correlations						Exploratory correlations
	Average (1)	Equal (2)	Higher (3)	Lower (4)	AI (5)	SDO (6)	Altruism (7)
1	1	0.792**	0.742**	0.811**	0.531**	−0.387**	0.215*
2		1	0.547**	0.674**	0.136	−0.402**	−159
3			1	0.487**	0.101	−0.319*	0.045
4				1	0.196	−0.281*	0.243*
5					1	−0.131	0.169
6						1	−0.114
7							1

Confirmatory correlations were Bonferroni–Holm adjusted; exploratory correlations were not adjusted, ^∗^*p* ≤ 0.05, **p ≤ 0.001.

## Discussion

In the present study, we investigated the influence of social status and its relationship with social dominance orientation on behavior in a modified dictator game. Therefore, we let the participants play the role of the dictator against three socially hierarchical identities and one identity represented by artificial intelligence. We could show that with regard to social status, AI received less money compared to all social interaction partners, and partners of socially equal status profited the most. Moreover, a stronger social dominance orientation generally led to lower dictator offers and did not have a significant effect on the social and non-social levels.

Contrary to our hypotheses and the findings of Rodrigues et al. (2015), for example, dictators did not distribute money profitably for higher hierarchical levels and were not deficient for lower ones. A possible explanation for the differences is that in our study, status is translated with attributes that are also relevant at the educational and career level. Given that, for example, Rodrigues et al. (2015) used income as a proxy for social status, the different meanings of social status (monetary versus educational) might be the reason why we did not find the expected effects on dictators’ offers to hierarchical recipients. However, the exploratory correlation of altruism and the identity of lower status has shown that only those individuals who supposedly need more support benefit from higher dictator offers. This coincides with the findings of Rodrigues et al. (2015) and supports the validity of the paradigm presented here. The fact that in our paradigm the largest amount of money was given to recipients of the same status as the dictator is consistent with findings on ingroup favoritism, according to which in cooperative tasks members of one’s own group are treated more favorably than outgroup members or strangers (e.g., Balliet et al., 2014). In terms of social status independent of SDO, there is a perception of AI that differs from the social identities, which is manifested in the low dictator offers. However, from a rational point of view, it makes no sense at all to give any money to AI. Therefore, the mean offer of 1.32€ is surprisingly high. One explanation would be that in a student sample of psychology students, there might be a response bias, especially in relation to the effect of AI. Our finding contradicts, at least in part, the findings of Melo et al. (2016) who found that humans have no sense of guilt when they exploit machines. Several studies have already shown that there are behavioral differences in interactions with machines compared to humans in tasks requiring social decision making (e.g., Gallagher et al., 2002). At the neural level, Gallagher et al. (2002) could show that when interacting with machines compared to humans in a rock–paper–scissors game, the medial prefrontal cortex was not particularly activated, an area that is associated with mentalizing (assumptions of a counterpart’s intentions and beliefs). In order to break down these differences in the way people handle machines more precisely, future studies could take a closer look at individual differences that might shed light on supposedly irrational generous behavior toward machines.

In our sample, SDO was associated with lower monetary offers to all hierarchical levels (i.e., AI, equal, higher, and lower). In the context of this study, this would mean that individuals who consider themselves more socially dominant generally want to gain a financial advantage over other human individuals. Thus, one could speak of ubiquitous dominance, which applies regardless of the social status of the interaction partner. This result contrasts with previous findings, which have shown that higher dominance is associated with a devaluation of individuals with lower status (Umphress et al., 2008). In addition, it was found that people with a highly developed SDO prefer to surround themselves with members of groups with socially high status, i.e., they favor high social status (Umphress et al., 2007). Possible missing interaction effects could also be due to our sample size, since social dominance at least tends to interact with the contrast human versus AI. The *post hoc* comparison of correlation coefficients supports this trend, since above all the correlations between SDO and the social identities are substantially different from the correlation between SDO and monetary offers to AI.

Also, one might expect that there is still a difference between the constructs of social dominance and egoism/altruism. As indicated by the exploratory correlations, altruism and dominance indicate opposing dictator decisions, as dominance led to less and altruism to more generous offers. This coincides with the finding that SDO is negatively correlated with cooperation and positively correlated with the tendency to behave selfishly with resources in economic games (e.g., Halali et al., 2018). While social dominance may be linked to low altruism, the findings of Rodrigues et al. (2015) were derived with extreme group selection, while in the case of the present study, only an *ad hoc* sampling was done. Therefore, it is still questionable whether this sampling has led to a similar distribution of the construct to actually compare the findings.

We briefly want to discuss several limitations of the present study. First, the present results are based primarily on female participants. Previous studies suggest that women act more altruistically compared to men in the dictator game (Rand et al., 2016; Brañas-Garza et al., 2018). Thus, the fact that socially higher and lower ranked recipients receive the same amount and AI receives anything at all could also be a mere gender effect. This is also reflected in the limited variance and distribution of SDO in the present sample (*M* = 2.62, *SD* = 0.83). Furthermore, we have a rather small sample size with regard to the investigation of individual differences. As debated, for example, by Schönbrodt and Perugini (2013); Gelman and Carlin (2014), and Patil et al. (2016), too small samples are problematic when it comes to the reproducibility of empirical findings. With regard to the individual differences in SDO, both aspects are the reason why this study has the character of a pilot study, which requires a larger and more sophisticated sample for a detailed evaluation. Additionally, there are empirical findings showing that there is no general statistically significant difference between hypothetical and real rewards, although individuals with certain traits (e.g., agreeableness and extraversion) behave differently when receiving real compared to hypothetical rewards (Ben-Ner et al., 2008). Other studies indicate differences between hypothetical and real rewards based on the mechanics of the game. While the first round of a repeated ultimatum game did not show any difference, from the second round on, players offered less hypothetical money (Gillis and Hettler, 2007). When playing Andreoni’s Public Goods Game, however, the authors could not find a substantial difference in behavior between hypothetical and real rewards. Thus, future studies might also examine the influence of hypothetical versus real monetary offers on the behavior of socially dominant individuals. Finally, future studies might counterbalance the order of the SDO measure and the dictator game. Filling in the SDO questionnaire first might have made salient social hierarchy to the participants.

In summary, the presented study showed that bargaining with an artificial intelligence seems to be conceptually different from the interaction with social identities, which opens new perspectives on human–machine interactions for future studies. Social dominance orientation predicted a more selfish behavior in a socially dominant position, regardless of the social rank of the interaction partner. In line with the trait activation theory (Tett and Burnett, 2003), the DG provided the necessary trait-relevant dominance gradient to evoke dominant behavior.

## Data Availability Statement

The datasets generated for this study are available on request to the corresponding author.

## Ethics Statement

Ethical review and approval was not required for the study on human participants in accordance with the local legislation and institutional requirements. The patients/participants provided their written informed consent to participate in this study.

## Author Contributions

MW designed and performed the experiments. MW, JR, and MP analyzed the data. MW wrote the manuscript in consultation with JR, MP, and JH. All authors contributed to the article and approved the submitted version.

## Conflict of Interest

The authors declare that the research was conducted in the absence of any commercial or financial relationships that could be construed as a potential conflict of interest.
